# Direct characterization of a nonlinear photonic circuit’s wave function with laser light

**DOI:** 10.1038/lsa.2017.143

**Published:** 2018-01-12

**Authors:** Francesco Lenzini, Alexander N Poddubny, James Titchener, Paul Fisher, Andreas Boes, Sachin Kasture, Ben Haylock, Matteo Villa, Arnan Mitchell, Alexander S Solntsev, Andrey A Sukhorukov, Mirko Lobino

**Affiliations:** 1 Centre for Quantum Dynamics, Griffith University, Brisbane, QLD 4111, Australia; 2 Nonlinear Physics Centre, Research School of Physics and Engineering, Australian National University, Canberra, ACT 2601, Australia; 3 ITMO University, Saint Petersburg 197101, Russia; 4 Ioffe Institute, Saint Petersburg 194021, Russia; 5 School of Engineering, RMIT University, Melbourne, VIC 3000, Australia; 6 School of Mathematical and Physical Sciences, University of Technology Sydney, Ultimo, NSW 2007, Australia; 7 Queensland Micro- and Nanotechnology Centre, Griffith University, Brisbane, QLD 4111, Australia

## Abstract

Integrated photonics is a leading platform for quantum technologies including nonclassical state generation^1, 2, 3, 4^, demonstration of quantum computational complexity^5^ and secure quantum communications^6^. As photonic circuits grow in complexity, full quantum tomography becomes impractical, and therefore an efficient method for their characterization^7, 8^ is essential. Here we propose and demonstrate a fast, reliable method for reconstructing the two-photon state produced by an arbitrary quadratically nonlinear optical circuit. By establishing a rigorous correspondence between the generated quantum state and classical sum-frequency generation measurements from laser light, we overcome the limitations of previous approaches for lossy multi-mode devices^9, 10^. We applied this protocol to a multi-channel nonlinear waveguide network and measured a 99.28±0.31% fidelity between classical and quantum characterization. This technique enables fast and precise evaluation of nonlinear quantum photonic networks, a crucial step towards complex, large-scale, device production.

Practical applications of quantum photonic technologies^11, 12^ require the integration of linear and nonlinear waveguides on a single device, where photons can be generated^1, 4^ and manipulated^13^. Spontaneous parametric down-conversion (SPDC) and spontaneous four-wave mixing are the two most common processes used for photon generation on chip with the former being the most efficient by far, needing only a few microwatts of pump power for generation rates exceeding several MHz^14, 15^. Monolithic integration of SPDC sources with multi-port optical circuits has been achieved in several contexts, with applications in quantum communication^16^, quantum metrology^1^, spatial multiplexing of heralded single-photon sources^17^, quantum state generation in nonlinear waveguide arrays^2^ and small-scale reconfigurable quantum photonic circuits^18^.

The near future of quantum photonics will involve an expansion in scale and applications of integrated circuits. However, the characterization of the two-photon state generated by a nonlinear waveguide network is a cumbersome experimental task^19^, requiring the collection of statistics from coincidence counts and a quadratically increasing number of measurements with system size. Here we propose and demonstrate a practical method for the characterization of the two-photon wavefunction generated by an arbitrary device with quadratic nonlinearity that uses only laser probes and power measurements. This technique fully reconstructs the spectral and spatial properties of the generated photon pairs from the measurements of bright optical beams and, with optimized hardware, it performs the same number of measurements at least four orders of magnitude faster than the corresponding quantum characterization. Our protocol is of both fundamental and practical importance for the development of integrated quantum photonics technologies including characterization of large-scale wafer production.

A method based on stimulated emission tomography (SET) was proposed^9^ for predicting the two-photon wavefunction produced by a nonlinear device using the analogy between spontaneous nonlinear processes and their classical stimulated counterparts, that is, difference-frequency generation or stimulated four-wave mixing. This technique was demonstrated for spectral characterization of two-photon states^20, 21, 22, 23^, and fast reconstruction of the density matrix of entangled-photon sources^24, 25^.

However, SET has never been realized on multimode optical networks since it requires injection of the seed beam into the individual supermodes supported by the structure^26^. A possible workaround is to inject the seed beam into each single channel individually then perform a transformation through supermode decomposition to obtain quantum predictions. Regardless, complete knowledge of the linear light dynamics inside the whole structure is required, making SET a multi-step procedure prone to errors and not applicable to ‘black-box’ circuits. Additionally, SET is strictly valid only in the limit of zero propagation losses^10^, posing a fundamental limitation for the characterization of real optical circuits.

Characterization via sum-frequency generation (SFG), the reverse process of SPDC, gives exact results in the presence of any type of losses. This approach was previously formulated only for single, homogeneous waveguides^10^, posing a stringent restriction for the characterization of more complex devices. In this work, we uncover a fundamentally important equivalence between the biphoton wavefunction and the classical sum-frequency field generated in the reverse direction of SPDC for any multimode non-linear device, overcoming the limitations of previous approaches. Our theoretical analysis is based on the rigorous use of the Green-function method^27^ (Supplementary Information), and holds for arbitrarily complex second-order nonlinear circuits, in the presence of any type of losses. More importantly, the SFG-SPDC analogy can be expressed in any measurement basis, providing a simple and fast experimental tool for the characterization of any ‘black-box’ *χ*^2^ -nonlinear process (Figure 1).

Multimode SFG characterization can reconstruct any degree of freedom of the photonic state including spatial mode, frequency, time-bin, and polarization. Here we illustrate its application to a ‘black-box’ device with N spatial modes of the same polarization, as schematically depicted in Figure 1. When a pump beam with frequency *ω*_p_ is injected into waveguide *n*_*p*_ at the input of the device it produces, by SPDC, the biphoton state (Figure 1a)


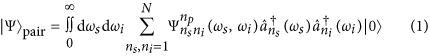


where *n*_*s*_(*n*_*i*_) is the index for signal(idler) output waveguide number, 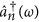 is the photon creation operator in the waveguide *n* with the frequency *ω*, and 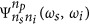 is the two-photon wavefunction^20^. In the classical SFG process shown in Figure 1b, two beams with signal frequency *ω*_*s*_ and idler frequency *ω*_*i*_ are injected into waveguides *n*_*s*_ and *n*_*i*_ from the SPDC output directions. The generated sum-frequency electric field 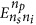 is detected from waveguide *n*_*p*_.

We reveal that the sum-frequency field in the undepleted pump regime is directly proportional to the two-photon wavefunction 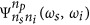 (Supplementary Information). From this correspondence we infer the squared amplitudes of the wavefunction elements by direct optical measurements of the sum-frequency power *P*_*SFG*_, and predict the absolute photon-pair generation rates for SPDC through the relation:


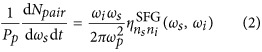


Here, *P*_p_ is the pump beam power, d*N*_pair_/d*ω*_*s*_d*t* is the photon-pair generation rate per unit signal frequency, and 
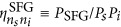
 is the sum-frequency conversion efficiency. Full spectral characterization of the biphoton state is obtained by scanning the signal and idler wavelengths, with an accuracy that is limited only by the spectral resolution of the laser source. In addition we can characterize the relative phases of the wavefunction components by classical interferometric measurements of the generated sum-frequency field.

The validity of the SFG protocol for multimode and inhomogeneous circuits was experimentally verified on an array of three evanescently coupled nonlinear waveguides schematically depicted in Figure 2a. The device was fabricated in lithium niobate by the use of the Reverse Proton Exchange technique^28, 29^ and heated to *T*=84 °C to obtain phase matching at *λ*=1550 nm. The waveguides have an inhomogeneous and asymmetric poling pattern along the propagation direction in order to test the generality of the method where laser light propagates in the opposite direction of the SPDC process (Supplementary Information).

We performed the SFG measurements by coupling two frequency tunable lasers into the device and measuring sum-frequency generation from waveguide 1. Figure 2b shows the SFG efficiency *η*_*SFG*_ as a function of signal and idler wavelengths coupled to the waveguides 2 and 3, respectively. Similar data were taken for all input combinations (Supplementary Fig. S1).

Figure 2c shows the probabilities 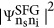 predicted from SFG efficiencies as a function of the SPDC pump wavelength for the degenerate case *λ*_s_=*λ*_i_=2*λ*_p_. Similar results are predicted for nondegenerate SPDC since the phase-matching bandwidth of the device is larger than the tuning range of our laser (Figure 2b).

We verified our characterization results by measuring the biphoton state generated when a *λ*_*p*_=775 nm pump is coupled into waveguide 1 in the reverse direction and the down-converted photon pairs pass through a 6 nm band-pass filter (Supplementary Fig. S2). Figure 2d shows two characteristic time histograms of photon coincidences for outputs from the waveguides 2–3 and 1–2 acquired by two avalanche photodiodes and a time tagging module. Coincidence-to-accidental-ratio (CAR) is ~24.5.

Figure 2e shows the squared amplitudes of the wavefunction elements predicted by SFG and those directly measured through normalization of SPDC coincidences (see complete data set and speed up analysis in Supplementary Information, and Materials and Methods for details on the calculation). SFG predictions are obtained by integrating the measured conversion efficiencies over a bandwidth of 6 nm along the diagonal 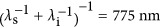. The two matrices have a fidelity 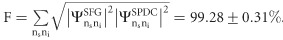. From equation (2), using the SFG measurements, we calculated a photon pair generation rate *N*_SFG_=2.36±0.14 MHz, which is the sum of the rates from all 6 output combinations. Direct measurement of this rate from SPDC data gives *N*_SPDC_=1.67±0.15 MHz, showing a good qualitative agreement between the two values. We believe that an overestimation of the detector efficiencies from the *η*_1_=8% and *η*_2_=10% provided by the manufacturer, and not measurable with our current setup, introduces a systematic error that underestimates the measured SPDC rate.

Our method allows direct characterization of the phases between the wavefunction elements, by performing interferometric detection of the generated sum-frequency field. Verification of the generated state by quantum state tomography would be experimentally difficult due to phase fluctuations between the different paths introduced by thermal and mechanical instabilities. Hence, the SFG-phase measurements are presented as a proof-of-concept and not directly verified by SPDC measurements.

Figure 3a shows the phase measurements setup used for input into waveguides 2 and 3. This procedure allows us to infer the relative phases between wavefunction elements 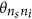 up to the phases of signal and idler beams 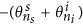 from the output of waveguide 1 (Figure 3b). The predicted wavefunction phases are shown in Figure 3c. Since the unknown phase multiplier 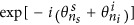 does not alter the degree of entanglement of the biphoton state, we calculated a Schmidt number^30^ *S*=1.59. (Supplementary Information), which precisely characterizes the degree of spatial entanglement and cannot be obtained with only photon correlations.

The SFG characterization method proposed here provides a practical path for characterization and development of monolithically integrated networks that for devices similar to ours can be four orders of magnitude faster than the equivalent quantum measurements and with two orders of magnitude greater accuracy (Supplementary Information). This technique can be applied to any arbitrary ‘black-box’ second-order nonlinear device and supports the development of integrated photon sources and large-scale quantum photonics technologies. In the future it will be of interest to explore how the SFG analogy can be applied to larger photon number states generated through SPDC.

## Materials and methods

### Experimental setup for SFG measurement

Signal and idler beams, generated by two tunable laser diodes with 100 kHz linewidth, were injected into each pair of waveguides with a fibre V-groove array. All the beams were collected in free-space at the output of the waveguides with a lens with 0.5 NA. SFG and signal-idler wavelengths were separated with a dichroic mirror. SFG power from the output of waveguide 1 and signal-idler powers from the outputs of all three waveguides were then measured with two standard power meters. The measured powers were corrected for Fresnel losses at the chip interface and used to calculate the SFG conversion efficiency at the output of the array. SFG conversion efficiencies for the single channel inputs were measured by combining signal and idler beams with a 50:50 fibre coupler. The measurement process was automated with Labview.

### Experimental setup for SPDC measurements

A pump beam with 775 nm wavelength and 100 kHz linewidth was generated by second-harmonic generation in a periodically poled lithium niobate waveguide and injected into waveguide 1 with a lens of 0.5 NA. The three outputs were collected with a fibre V-groove array, and photon coincidences between each pair of waveguides were measured with two gated InGaAs avalanche photodiodes and a time-tagging module. A filtering stage in free-space, made from a set of 5 long-pass filters and a band-pass filter, was used to attenuate the pump beam by 150 dB. Photon pairs were filtered with a 6 nm band-pass filter centred at *λ*_*c*_=1550 nm to restrict the SPDC emission bandwidth to the range measured by SFG. Photon coincidences from the single channels were measured by splitting signal-idler photons with a 50:50 fibre coupler.

### Absolute photon pair generation rates and relative squared amplitudes of the wavefunction from SFG measurements

For each pair of waveguides *n*_*s*_, *n*_*i*_ the signal wavelength was scanned in steps of Δ*λ*=0.25 nm in a 6 nm bandwidth centered at 1550 nm. At each step *j* the idler wavelength was set to 
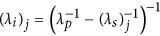
, where *λ*_p_=775 nm is the pump wavelength for SPDC. Absolute photon pair generation rates were calculated by discretization of equation (2) through the relation


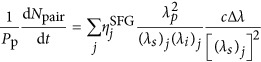


where 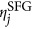 is the normalized sum-frequency conversion efficiency measured at each step *j*. The pump power *P*_*p*_ was measured during the SPDC characterization from the first output of the fibre array. Relative squared amplitudes of the wavefunction elements were calculated as


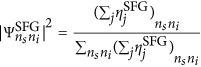


### Error in the fidelity between correlation matrices

The error in the fidelity between the correlation matrices predicted by SFG and measured by SPDC was calculated with an iterative numerical algorithm with *N*=10^6^ cycles. At each step we assigned to the two correlation matrices a random value calculated from a normal distribution with a sigma given by the error in the measurements. Average value and error in the fidelity were finally calculated from the simulated distribution.

### Second-harmonic generation contributions in SFG measurements

For SFG-power measurements second-harmonic generation (SHG) contributions were first measured by inputting signal and idler beams into each channel individually. SHG powers were then subtracted from SFG-power measurements. The procedure was repeated and automated with Labview. For SFG-phase measurements, SFG and SHG contributions were separated at the output of the array with the aid of a diffraction grating.

## Author contributions

ANP, JT, ASS and AAS developed the theory for the SFG-SPDC analogy. FL, AB, SK and BH fabricated the nonlinear device. FL, PF, AB and MV performed the experimental measurements. AM, ASS, AAS and ML supervised the project. FL wrote the manuscript with contributions from all authors.

## Figures and Tables

**Figure 1:**
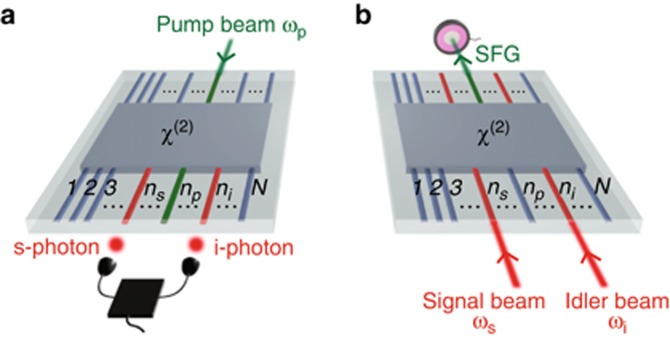
Scheme for the characterization of the biphoton state produced by an array of N waveguides with an arbitrary *χ*^(2)^-nonlinear process. (**a**) SPDC: a pump beam is injected into waveguide *n*_*p*_ at the input of the device. Photon-coincidence counting measurements between each pair of waveguides (*n*_*s*_, *n*_*i*_) at the output are used to measure photon-pair generation rates and relative absolute squared values of the wavefunction. (**b**) SFG: Laser light at signal and idler frequencies is injected into waveguides *n*_*s*_ and *n*_*i*_ in the reverse direction of SPDC. Absolute photon-pair generation rates and relative absolute squared values of the wavefunction can be predicted by direct optical power detection of the sum-frequency field emitted from waveguide *n*_*p*_.

**Figure 2:**
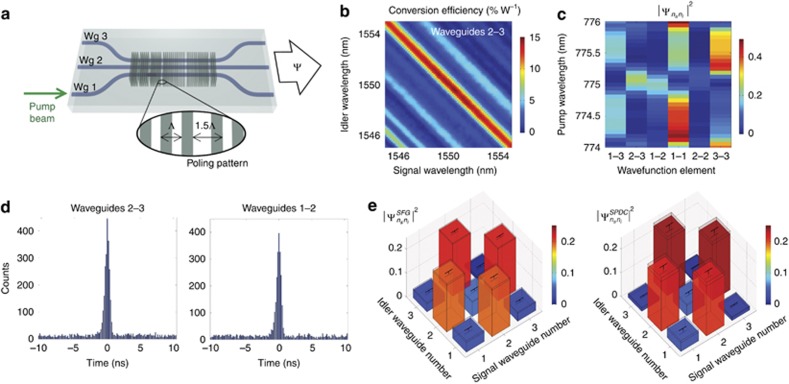
Comparison between SFG and SPDC measurements. (**a**) Schematic of the device used for biphoton state generation. The device is made of three coupled waveguides with five defects in the poling pattern introduced by translating the poled domains by half a poling period Λ (inset). This design is based on the recently developed concept for quantum state engineering with specialized poling patterns^26^. Waveguides are fabricated on a lithium niobate substrate by reverse proton exchange (Supplementary Information)^28, 29^. (**b**) Measured classical sum-frequency conversion efficiency from waveguide 1 as a function of signal and idler wavelengths coupled to waveguides 2 and 3. (**c**) Predicted squared relative amplitudes of the biphoton wavefunction for a pump injected into waveguide 1, proportional to the SFG signal for different combinations of signal and idler in coupled waveguides vs. the pump wavelength in the degenerate regime (*λ*_*s*_=*λ*_*i*_=2*λ*_*p*_). (**d**) Time histogram for the photon coincidences between waveguides 2–3 and waveguides 1–2 for a 50 min acquisition time, a pump wavelength *λ*_p_=775 nm, and a pump power *P*_p_=32±5 μW. Time bin width is 82 ps. Complete data sets are in Supplementary Fig. S2. (**e**) Normalized biphoton wavefunctions predicted by SFG (left) and measured by SPDC (right) for *λ*_*p*_=775 nm.

**Figure 3:**
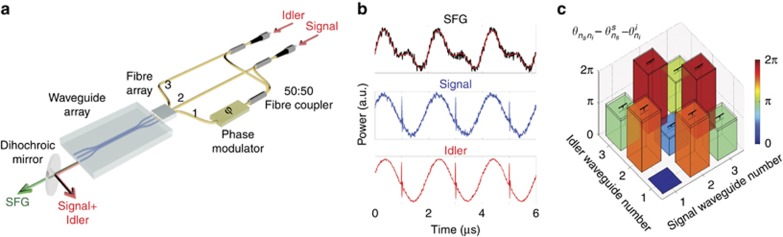
Measurement of the relative phases between wavefunction elements by SFG. (**a**) Schematic of the experimental setup for inputs into waveguides 2 and 3. Signal and idler beams are split and recombined with a network of 50:50 fibre couplers and injected into the three waveguides with a fibre V-groove array. An electro-optic phase modulator is used to generate an interference pattern between the sum-frequency fields generated from the combinations of signal and idler beams in waveguides 2–3 and waveguides 1-1. SFG and signal-idler beams are collected in free-space at the output of waveguide 1 with a lens of 0.5 NA (not shown in the figure) and separated with a dichroic mirror. A wavelength-division multiplexer (not shown in the figure) is used to separate signal and idler wavelengths. (**b**) Oscilloscope traces obtained by collecting the beams with three different photodiodes for a modulation frequency *f*=500 kHz. The three traces are used to measure the relative phase between the wavefunction elements Ψ_23_, Ψ_11_. Solid red line is the theoretical fit (see Supplementary Information for details). (**c**) Relative phases between wavefunction elements measured for all the combinations of signal-idler beams in the three waveguides. Waveguide 1 is the fixed reference for all the phase measurements. Measurements are performed for a signal wavelength *λ*_*s*_=1550.12 nm and an idler wavelength *λ*_*i*_=1556.65 nm. The sample was heated up to T=108 °C to get a phase matching condition centred at 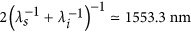. See Supplementary Information for a calculation of the error bars.
