# Supplementary material for: Direct characterization of a nonlinear photonic circuit’s wave function with laser light
Source: Light Sci Appl. 2018 Jan 12;7:17143–. doi: 10.1038/lsa.2017.143 (PMC6107051; doi:10.1038/lsa.2017.143)
Supplement: Supplementary Information [file lsa2017143x1.docx]

| **WAVEGUIDE INPUT** | **MEASURED SPLITTING RATIOS** | **COUPLED MODE THEORY** |  |
| --- | --- | --- | --- |
| 1 | OUT1= 7.8%  OUT2= 8.2%  OUT3= 84.1% | OUT1= 0.1%  OUT2= 7.9%  OUT3= 91.9% |  |
| 2 | OUT1= 2.3%  OUT2= 87.0%  OUT3= 10.7% | OUT1= 7.9%  OUT2= 84.2%  OUT3= 7.9% |  |
| 3 | OUT1= 87.8%  OUT2= 4.1%  OUT3= 8.2% | OUT1= 91.9%  OUT2= 7.9%  OUT3= 0.1% |  |
