# Supplementary material for: Direct characterization of a nonlinear photonic circuit’s wave function with laser light
Source: Light Sci Appl. 2018 Jan 12;7:17143–. doi: 10.1038/lsa.2017.143 (PMC6107051; doi:10.1038/lsa.2017.143)
Supplement: Supplementary Information [file lsa2017143x2.docx]

| **WF Element** | **SPDC** | **SFG** |
| --- | --- | --- |
| $\left\vert\psi_{13} \right\vert^{2},\left\vert\psi_{31} \right\vert^{2}$ | $0.013\pm0.003$ | $0.033\pm0.005$ |
| $\left\vert\psi_{23} \right\vert^{2},\left\vert\psi_{32} \right\vert^{2}$ | $0.229\pm0.013$ | $0.218\pm0.005$ |
| $\left\vert\psi_{12} \right\vert^{2},\left\vert\psi_{21} \right\vert^{2}$ | $0.212\pm0.015$ | $0.195\pm0.007$ |
| $\left\vert\psi_{11} \right\vert^{2}$ | $0.040\pm0.010$ | $0.040\pm0.008$ |
| $\left\vert\psi_{22} \right\vert^{2}$ | $0.046\pm0.007$ | $0.059\pm0.005$ |
| $\left\vert\psi_{33} \right\vert^{2}$ | $0.006\pm0.002$ | $0.009\pm0.004$ |
