# Supplementary material for: Direct characterization of a nonlinear photonic circuit’s wave function with laser light
Source: Light Sci Appl. 2018 Jan 12;7:17143–. doi: 10.1038/lsa.2017.143 (PMC6107051; doi:10.1038/lsa.2017.143)
Supplement: Supplementary Material [file lsa2017143x3.docx]

**Supplementary Material for**

**Direct characterization of a nonlinear photonic circuit's wave function with laser light**

Francesco Lenzini^1^, Alexander N. Poddubny^2,3,4^ , James Titchener^2^,

Paul Fisher^1^ , Andreas Boes^5^ , Sachin Kasture^1^ , Ben Haylock^1^ , Matteo Villa^1^

Arnan Mitchell^5^ , Alexander S. Solntsev^2,6^ , Andrey A. Sukhorukov^2^ , and Mirko Lobino^1,7,^*

*^1^ Centre For Quantum Dynamics, Griffith University, Brisbane QLD 4111, Australia*

*^2^ Nonlinear Physics Centre, Research School of Physics and Engineering,*

*Australian National University, Canberra ACT 2601, Australia*

*^3^ ITMO University, Saint Petersburg 197101, Russia*

*^4^ Ioffe Institute, Saint Petersburg 194021, Russia*

*^5^ School of Engineering, RMIT University, Melbourne VIC 3000, Australia*

*^6^School of Mathematical and Physical Sciences, University of Technology Sydney, Ultimo NSW 2007, Australia*

*^7^ Queensland Micro- and Nanotechnology Centre, Griffith University, Brisbane QLD 4111, Australia*

*** [*m.lobino@griffith.edu.au*](mailto:m.lobino@griffith.edu.au)

## **SPDC-SFG correspondence for a general “black-box”** $\boldsymbol{\chi}^{\boldsymbol{(2)}}$**-nonlinear process**

Here we present a derivation of the correspondence between spontaneous parametric down conversion (SPDC) process and the sum frequency generation (SFG) process in the reversed geometry for an arbitrary $\chi^{(2)}$-nonlinear structure, that is reciprocal in the linear regime. This proof generalizes the Lorentz reciprocity theorem^1^ in the form

|  | $\int\text{d}^{3}r\boldsymbol{P}_{1}\left( \boldsymbol{r} \right)\cdot\boldsymbol{E}_{2}\left( \boldsymbol{r} \right)=\int\text{d}^{3}r\boldsymbol{P}_{2}\left( \boldsymbol{r} \right)\cdot\boldsymbol{E}_{1}\left( \boldsymbol{r} \right)$ | (S1) |
| --- | --- | --- |
|  |  |  |

that links the electric field distribution $\boldsymbol{E}_{1}(\boldsymbol{r})$ and $\boldsymbol{E}_{2}(\boldsymbol{r})$, induced by the polarization distributions $\boldsymbol{P}_{1}$ and $\boldsymbol{P}_{2}$, respectively.

The biphoton wavefunction in the SPDC regime^2^ reads

|  | $\Psi\left( \boldsymbol{r}_{s},\boldsymbol{r}_{i},\sigma_{s},\sigma_{i},\omega_{s},\omega_{i} \right)=\int\text{d}^{3}r_{0}G_{\sigma_{s}\alpha}\left( \boldsymbol{r}_{s},\boldsymbol{r}_{0};\omega_{s} \right)G_{\sigma_{i}\beta}\left( \boldsymbol{r}_{i},\boldsymbol{r}_{0};\omega_{i} \right)\chi_{\alpha\beta\gamma}^{\left( 2 \right)}E_{p,\gamma}\left( \boldsymbol{r}_{0} \right) ,$ | (S2) |
| --- | --- | --- |
|  |  |  |

where $G$ is the electromagnetic tensor Green function satisfying the equation

|  | $\left[ \text{rot rot}-\left( \frac{\omega}{c} \right)^{2}\varepsilon\left( \boldsymbol{r} \right) \right]\hat{G}\left( \boldsymbol{r},\boldsymbol{r}^{\boldsymbol{'}};\omega\right)=4\pi\left( \frac{\omega}{c} \right)^{2}\delta\left( \boldsymbol{r}-\boldsymbol{r}^{\boldsymbol{'}} \right) ,$ | (S3) |
| --- | --- | --- |

where $\boldsymbol{E}_{p}$ is the pumping wave and $\chi^{(2)}$ is the nonlinear susceptibility tensor. The indices $\sigma_{s}$ and $\sigma_{i}$ label signal and idler polarizations, respectively. Summation over dummy Cartesian indices $\alpha, \beta,\gamma=x,y,z$ is assumed.

On the other hand, the nonlinear wave at the sum frequency $\omega_{p}=\omega_{s}+\omega_{i}$ generated from the waves $\boldsymbol{E}_{s}\left( \boldsymbol{r}_{s} \right), \boldsymbol{E}_{i}\left( \boldsymbol{r}_{i} \right)$ in the nonlinear structure can be presented as

|  | $E_{SFG,\sigma_{p}}\left( \boldsymbol{r}_{p} \right)=\int\text{d}^{3}r_{0}G_{\sigma_{p}\gamma}\left( \boldsymbol{r}_{p},\boldsymbol{r}_{0} \right)\chi_{\alpha\beta\gamma}^{\left( 2 \right)}\left( \boldsymbol{r}_{0} \right)E_{s,\alpha}\left( \boldsymbol{r}_{0} \right)E_{i,\beta}\left( \boldsymbol{r}_{0} \right) .$ | (S4) |
| --- | --- | --- |
|  |  |  |

Inspired by linear reciprocity relationship Eq. (S1) we introduce the polarizations $\boldsymbol{P}_{s,i,p}(\boldsymbol{r})$ inducing the corresponding waves $\boldsymbol{E}_{s,i,p}(\boldsymbol{r})$,

|  | $\boldsymbol{E}_{\nu}\left( \boldsymbol{r} \right)=\int\text{d}^{3}r_{0}\hat{G}\left( \boldsymbol{r},\boldsymbol{r}_{0};\omega_{p} \right)\boldsymbol{P}_{\nu}\left( \boldsymbol{r}_{0} \right), \nu=s,i,p .$ | (S5) |
| --- | --- | --- |
|  |  |  |

This allows us to rewrite Eq. (S2) and Eq. (S4) as

|  | $\Psi\left( \boldsymbol{r}_{s},\boldsymbol{r}_{i},\sigma_{s},\sigma_{i},\omega_{s},\omega_{i} \right)=\int\text{d}^{3}r_{0}\int\text{d}^{3}r_{p}G_{\sigma_{s}\alpha}\left( \boldsymbol{r}_{s},\boldsymbol{r}_{0} \right)G_{\sigma_{i}\beta}\left( \boldsymbol{r}_{i},\boldsymbol{r}_{0} \right)\chi_{\alpha\beta\gamma}^{\left( 2 \right)}\left( \boldsymbol{r}_{0} \right)G_{{\gamma\sigma}_{p}}(\boldsymbol{r}_{0},\boldsymbol{r}_{p})P_{p,\sigma_{p}}(\boldsymbol{r}_{p}) ,$ | (S6) |
| --- | --- | --- |
|  |  |  |

and

|  | $E_{SFG,\sigma_{p}}\left( \boldsymbol{r}_{p};\omega_{i}+\omega_{s} \right)=\int\text{d}^{3}r_{0}\int\text{d}^{3}r_{i}\int\text{d}^{3}r_{s}G_{\sigma_{p}\gamma}\left( \boldsymbol{r}_{p},\boldsymbol{r}_{0} \right)\chi_{\alpha\beta\gamma}^{\left( 2 \right)}\left( \boldsymbol{r}_{0} \right)G_{\alpha\sigma_{s}}\left( \boldsymbol{r}_{0},\boldsymbol{r}_{s} \right)G_{\beta\sigma_{i}}\left( \boldsymbol{r}_{0},\boldsymbol{r}_{i} \right)P_{s,\sigma_{s}}(\boldsymbol{r}_{s})P_{i,\sigma_{i}}(\boldsymbol{r}_{i}) .$ | (S7) |
| --- | --- | --- |
|  |  |  |

We have omitted the frequency arguments in the Green functions for the sake of brevity. In the reciprocal structure the Green functions satisfy the reciprocity property

|  | $G_{\alpha\beta}\left( r_{1},r_{2} \right)=G_{\beta\alpha}\left( r_{2},r_{1} \right) ,$ | (S8) |
| --- | --- | --- |
|  |  |  |

that is equivalent to Eq.(S1). Comparing Eq. (S6) and Eq. (S7) with the help of Eq. (S8) we establish the general reciprocity relationship between SPDC and SFG processes in the form

|  | $\iint\text{d}^{3}r_{i}\text{d}^{3}r_{s}\Psi\left( \boldsymbol{r}_{s},\boldsymbol{r}_{i},\sigma_{s},\sigma_{i},\omega_{s},\omega_{i} \right)P_{i,\sigma_{i}}(\boldsymbol{r}_{i})P_{s,\sigma_{s}}(\boldsymbol{r}_{s})=\int\text{d}^{3}r_{p}E_{SFG,\sigma_{p}}\left( \boldsymbol{r}_{p};\omega_{i}+\omega_{s} \right)P_{p,\sigma_{p}}(\boldsymbol{r}_{p}) .$ | (S9) |
| --- | --- | --- |
|  |  |  |

## **SPDC-SFG correspondence for a coupled waveguide array**

In the previous section we have presented a general proof of the SPDC-SFG correspondence for a reciprocal detection and excitation geometries. Here we apply this concept to the particular situation of an array of coupled waveguides, parallel to the *z* axis. The waveguides can have arbitrary mode dispersion and losses and can be periodically patterned.

**Green function expansion.** In what follows it will be useful to expand the Green function Eq. (S3) over the set of the Bloch eigenmodes of the structure with the Bloch wavenumbers $\beta_{n}$, characterizing propagation along *z* direction at the given frequency $\omega$. The polarization degree of freedom is included in the index *n* as well. Electric and magnetic fields of the eigenmodes satisfy the orthogonality relation^3,4^

|  | $\iint\text{d}x\text{d}y\left[ \boldsymbol{E}_{n'}\times\boldsymbol{H}_{n}-\boldsymbol{E}_{n}\times\boldsymbol{H}_{n'} \right]_{z}=s_{n}\delta_{n,-n'} ,$ | (S10) |
| --- | --- | --- |
|  |  |  |

where $s_{n}$ is the so-called adjoint flux and we denote by the index $-n$ the solution with the wave vector $-\beta_{n}$. A pair of such forward and backward propagating solutions exists at any frequency provided that the structure is reciprocal. The Green function can be sought as an expansion over the eigenmodes propagating away from the point $z'$:

|  | $G_{\mu\nu}\left( \boldsymbol{r},\boldsymbol{r}^{\boldsymbol{'}} \right)=\left\{ \begin{aligned} \sum_{\text{Im} \beta_{n}>0} u_{n,\nu}E_{n,\mu}(\boldsymbol{r}) &z>z' \\ \sum_{\text{Im} \beta_{n}<0} u_{n,\nu}E_{n,\nu}(\boldsymbol{r}) &z<z^{'} . \end{aligned} \right.$ | (S11) |
| --- | --- | --- |
|  |  |  |

Here we distinguish between right- and left-propagating modes $\propto e^{\pm i\beta z}$ by the sign of Im $\beta$; for a lossless medium an infinitely small loss can be formally added to the permittivity. In order to find the expansion coefficients $u_{n,\beta}$ we use the Lorentz reciprocity theorem in the form [3]

|  | $\frac{c}{4\pi}\oint_{S} \text{d}S\left( \boldsymbol{E}\times\boldsymbol{H}_{n'}-\boldsymbol{E}_{n'}\times\boldsymbol{H} \right)=-i\omega\int d^{3}r\boldsymbol{E}_{n^{'}}\cdot\boldsymbol{P} ,$ | (S12) |
| --- | --- | --- |
|  |  |  |

where $\boldsymbol{E}$ and $\boldsymbol{H}$ are the electric and magnetic fields induced by the dielectric polarization distribution $\boldsymbol{P}(\boldsymbol{r})$. Namely, we replace $\boldsymbol{E}$ in Eq. (S12) by the Green function expansion Eq. (S11) and $\boldsymbol{P}(\boldsymbol{r})$ by the point source term $\boldsymbol{e}_{\upsilon}\delta(\boldsymbol{r}-\boldsymbol{r}^{\boldsymbol{'}})$ where $\boldsymbol{e}_{\beta}$ is the unitary basis vector. The integral in the left-hand side of Eq. (S12) is evaluated with the help of the orthogonality relation Eq.(S10). This yields the equation for the coefficients $s_{-n}u_{-n,\upsilon}=-4\pi i\omega e_{\nu}\cdot\boldsymbol{E}_{m}(\boldsymbol{r}^{\boldsymbol{'}})/c$. Finding $u_{n,\upsilon}$ from this equation we present the Green function as

|  | $G_{\alpha\beta}\left( \boldsymbol{r},\boldsymbol{r}^{\boldsymbol{'}} \right)=\sum_{\text{Im} \beta_{n}>0} f_{n}E_{n,\alpha}(\boldsymbol{r}_{>})E_{-n,\beta}(\boldsymbol{r}_{<})$ | (S13) |
| --- | --- | --- |
|  |  |  |

where $f_{n}=-4\pi i\omega/(cs_{m})$ and $\boldsymbol{r}_{>}$ ($\boldsymbol{r}_{<}$) denotes one of the vectors $\boldsymbol{r}$, $\boldsymbol{r'}$ with greater (lesser) coordinate $z$.

**Proof of the SPDC-SFG correspondence.** The complex wavefunction of a photon pair, generated in a $\chi^{(2)}$-nonlinear structure within the SPDC process, has the amplitude^2^

|  | $T\left( \boldsymbol{r}_{s}\sigma_{s},\boldsymbol{r}_{i}\sigma_{i} \right)=\int\text{d}^{3}r_{0}G_{\sigma_{s}\nu}\left( \boldsymbol{r}_{s},\boldsymbol{r}_{0},\omega_{s} \right)G_{\sigma_{i}\mu}\left( \boldsymbol{r}_{i},\boldsymbol{r}_{0},\omega_{i} \right)\chi_{\mu\nu;\eta}^{\left( 2 \right)}\left( \boldsymbol{r}_{0} \right)E_{p,\eta}(\boldsymbol{r}_{0},\omega_{p}) ,$ | (S14) |
| --- | --- | --- |
|  |  |  |

where $\boldsymbol{r}_{s}$ ($\boldsymbol{r}_{i}$) and $\sigma_{s}$ ($\sigma_{i}$) are signal (idler) photon coordinates and polarizations, respectively, and $E_{p}$ is the electric field of the pump with the frequency $\omega_{p}$. We now assume that the structure is pumped in the eigenmode $n_{p}$, substitute the Green function in the form Eq. (S13) and rewrite the biphoton wavefunction in the eigenmode representation as

|  | $\Psi\left( n_{p}\to n_{s},n_{i} \right)=f_{n_{i}}f_{n_{s}}\int\text{d}^{3}r_{0}\chi_{\mu\nu,\eta}^{\left( 2 \right)}(\boldsymbol{r}_{0})E_{n_{p},\eta}(\boldsymbol{r}_{0})E_{{-n}_{s},\mu}(\boldsymbol{r}_{0})E_{{-n}_{i},\nu}(\boldsymbol{r}_{0}) .$ | (S15) |
| --- | --- | --- |
|  |  |  |

Now we consider the SFG process in the reverse direction. Two beams are injected into the “signal” and “idler” eigenmodes $-n_{s}$ and $-n_{i}$, propagating in the reverse direction. The generated SFG field is given by the convolution of the Green function with the nonlinear $\chi^{(2)}$-polarization induced by the incident waves,

|  | $E_{SFG,\sigma_{p}}\left( \boldsymbol{r} \right)=\int\text{d}^{3}r_{0}G_{\sigma_{p}\eta}\left( \boldsymbol{r},\boldsymbol{r}_{0} \right)\chi_{\mu\nu,\eta}^{\left( 2 \right)}\left( \boldsymbol{r}_{0} \right)E_{-n_{s},\mu}\left( \boldsymbol{r}_{0} \right)E_{-n_{i},\nu}(\boldsymbol{r}_{0}) .$ | (S16) |
| --- | --- | --- |
|  |  |  |

Substituting the Green function expansion Eq. (S13) into Eq. (S16) we find the dimensionless SFG conversion amplitude from the modes $-n_{s}$, $-n_{i}$ to the mode $-n_{p}$ propagating in the direction opposite to the pump of the SPDC process:

|  | $\xi\left( -n_{s},-n_{i}\to-n_{p} \right)=f_{n_{p}}\int\text{d}^{3}r_{0}\chi_{\mu\nu,\eta}^{\left( 2 \right)}\left( \boldsymbol{r}_{0} \right)E_{n_{p},\eta}(\boldsymbol{r}_{0})E_{{-n}_{s},\mu}(\boldsymbol{r}_{0})E_{{-n}_{i},\nu}(\boldsymbol{r}_{0}) .$ | (S17) |
| --- | --- | --- |
|  |  |  |

Comparing Eq. (S17) and Eq. (S15) we establish our main result, the correspondence between the biphoton wavefunction and the sum-frequency conversion efficiency

|  | $\Psi\left( n_{p}\to n_{s},n_{i} \right)=\frac{f_{n_{i}}f_{n_{s}}}{f_{n_{p}}}\xi\left( -n_{s},-n_{i}\to-n_{p} \right) .$ | (S18) |
| --- | --- | --- |
|  |  |  |

We stress that the exact structure of the eigenmodes $\boldsymbol{E}_{m}$ was never used in the proof. The only required property is the structure reciprocity in the linear regime, allowing to expand the Green function Eq. (S13) into the set of mutually reciprocal eigemodes $\boldsymbol{E}_{m}$ and $\boldsymbol{E}_{-m}$. As such, Eq. (S18) can be readily generalized to other geometries. The Green function can be expanded over the set of the solutions with the asymptotic of outgoing and incoming spherical waves. Once the proper set of reciprocal solutions is determined, the SPDC-SFG correspondence relation can be established.

**Predicting the absolute SPDC photon count rate from the SFG conversion efficiency**. The SFG power conversion efficiency (erg/sec) can be found from the conversion amplitude Eq. (S18) as

|  | $\eta_{n_{s}n_{i}}^{SFG}\left( \omega_{s},\omega_{i} \right)=\frac{\phi_{n_{p}}}{\phi_{n_{i}}\phi_{n_{s}}}\left\vert\xi\left( -n_{s},-n_{i}\to-n_{p} \right) \right\vert^{2} ,$ | (S19) |
| --- | --- | --- |
|  |  |  |

where $\phi_{n}=c \text{Re}\iint\text{d}x\text{d}y\boldsymbol{E}_{n}\times\boldsymbol{H}_{n}^{*}/(2\pi)$ is the energy flux for the mode $n$. In order to determine the photon coincidence count rate we need to calibrate the photon detection process [2]. To this end we explicitly introduce the signal and idler detectors modelled as the two-level systems with the dipole momenta matrix elements $\boldsymbol{d}_{i}, \boldsymbol{d}_{s}$ and the energies $\hbar\omega_{i}, \hbar\omega_{s}$. The number of photons absorbed by the detector per unit time is given by

|  | $\frac{\text{d}N_{\text{abs},i,s}}{\text{d}t}=\frac{2\pi}{\hbar}\delta\left( \hbar\omega-\hbar\omega_{i,s} \right)\left\vert\boldsymbol{d}_{i,s}\cdot\boldsymbol{E} \right\vert^{2} ,$ | (S20) |
| --- | --- | --- |
|  |  |  |

where $\boldsymbol{E}$ is the local electric field at the detector. On the other hand, the number of photons traveling through the given eigenmode $n_{i,s}$ per time is given by ${\text{d}N_{\text{phot}\text{,}\text{i,s}}}/{\text{d}t}={\phi_{n_{i,s}}}/{\hbar\omega_{i,s}}$. The ratio between the numbers of the absorbed photons and propagating photons provides the quantum efficiency of the detector for the mode $n_{i,s}$ ,

|  | ${QE}_{i,s}=\frac{N_{abs,i,s}}{N_{phot,i,s}}=\frac{2\pi\omega_{i,s}\left\vert\boldsymbol{d}_{i,s}\cdot\boldsymbol{E} \right\vert^{2}}{\hbar\phi_{n}} .$ | (S21) |
| --- | --- | --- |
|  |  |  |

The two-photon coincidence count rate per unit of the signal and idler spectra is formally defined as

|  | $\frac{\text{d}N_{pair}}{\text{d}t\text{d}\omega_{i}\text{d}\omega_{s}}=\frac{W_{is}}{QE_{i}QE_{s}}$ | (S22) |
| --- | --- | --- |
|  |  |  |

where

|  | $W_{is}=\frac{2\pi}{\hbar}\delta\left( \hbar\omega_{p}-\hbar\omega_{i}-\hbar\omega_{s} \right)\left\vert\sum_{\sigma_{i}\sigma_{s}} d_{i,\sigma_{i}}^{*}d_{s{,\sigma}_{s}}^{*}T\left( \boldsymbol{r}_{s}\sigma_{s},\boldsymbol{r}_{i}\sigma_{i} \right) \right\vert^{2},$ | (S23) |
| --- | --- | --- |
|  |  |  |

is the uncalibrated rate of two photon counts calculated from the bi-photon amplitude Eq.(S14) . In our geometry the generation and detection take place in the given eigenmodes $n_{s}$ and $n_{i}$. Substituting the definitions of the quantum efficiencies into Eq. (S22) we obtain

|  | $\frac{\text{d}N_{pair}}{\text{d}t\text{d}\omega_{i}\text{d}\omega_{s}}=\frac{\delta(\omega_{p}-\omega_{i}-\omega_{s})}{2\pi}\frac{\phi_{q_{i}}\phi_{q_{s}}}{\omega_{i}\omega_{s}}\left\vert\Psi\left( n_{p}\to n_{s},n_{i} \right) \right\vert^{2}\frac{P_{p}}{\phi_{q_{p}}}$ | (S24) |
| --- | --- | --- |
|  |  |  |

where $Pp$ (erg/sec) is the pump power (erg/sec). In order to compare the coincidence count rate Eq. (S24) with the sum frequency power conversion efficiency Eq. (S19) we make use of our general SPDC-SFG link Eq.(S18). This yields a general *absolute* correspondence between the sum frequency rate and the photon pair generation rate:

|  | $\frac{1}{P_{p}}\frac{\text{d}N_{pair}}{\text{d}t\text{d}\omega_{i}\text{d}\omega_{s}}=\frac{\delta\left( \omega_{p}-\omega_{i}-\omega_{s} \right)}{2\pi}\frac{\phi_{q_{i}}^{2}\phi_{q_{s}}^{2}\left\vert f_{q_{i}} \right\vert^{2}\left\vert f_{q_{s}} \right\vert^{2}}{\omega_{i}\omega_{s}\phi_{q_{p}}^{2}\left\vert f_{q_{p}} \right\vert^{2}}\eta_{n_{s}n_{i}}^{\text{SFG}}\left( \omega_{s},\omega_{i} \right) .$ | (S25) |
| --- | --- | --- |
|  |  |  |

We now assume that in the detection region the tunnelling coupling between the waveguides can be neglected and the flux and adjoint flux for each of the modes $n_{s}$, $n_{i}$, $n_{p}$ can be simplified to

|  | $\phi=\frac{c\mathcal{N}\left\vert E^{2} \right\vert S}{2\pi}, s=2S\left\vert E^{2} \right\vert\mathcal{N,}f=-\frac{2\pi i\omega}{cS\left\vert E^{2} \right\vert\mathcal{N}} ,$ | (S26) |
| --- | --- | --- |
|  |  |  |

where $S$ is a characteristic waveguide area and $\mathcal{N}$ is the dimensionless mode refractive index. The products $\phi f$ in Eq. (S25) then reduce to $\omega_{i}$, $\omega_{s}$, $\omega_{p}$, respectively, and the correspondence law Eq. (S25) assumes the simplified form

|  | $\frac{1}{P_{p}}\frac{\text{d}N_{pair}}{\text{d}t\text{d}\omega_{i}\text{d}\omega_{s}}=\frac{\delta\left( \omega_{p}-\omega_{i}-\omega_{s} \right)}{2\pi}\frac{\omega_{i}\omega_{s}}{\omega_{p}^{2}}\eta_{n_{s}n_{i}}^{\text{SFG}}\left( \omega_{s},\omega_{i} \right) .$ | (S27) |
| --- | --- | --- |
|  |  |  |

Integrating Eq. (S27) over the idler spectrum we recover Eq. (2) in the main text linking the two-photon count rate per unit signal frequency to the sum frequency generation rate.

**Speed-up of the SFG protocol over photon counting measurements**

We estimate the speedup of classical SFG vs. quantum SPDC device characterization, taking into account the effect of shot noise. Let us first consider a regime where the phase-matching bandwidth is much larger than the spectral filtering applied to the photons, and accordingly a single SFG measurement per output combination is sufficient for estimating the quantum behaviour of the device. Then, for the photon bandwidth $\Delta\omega_{s}$ and time $T_{SPDC}$ over which the photon-pair counts through SPDC are integrated, Eq. (2) can be written as

|  | $N_{pair}=P_{p}T_{SPDC}\Delta\omega_{s}\frac{\omega_{i}\omega_{s}}{2\pi\omega_{p}^{2}}{P_{SFG}}/{P_{s}P_{i}}.$ | (S28) |
| --- | --- | --- |

We then represent it as

|  | $N_{pair}\approx T_{SPDC}\frac{T_{p}}{{\tau_{s}N}_{p}}\frac{N_{SFG}}{T_{SFG}} \frac{P_{p}^{2}}{P_{s}P_{i}},$ | (S29) |
| --- | --- | --- |

where we take into account that $\omega_{i}{\approx\omega}_{s}{\approx\omega}_{p}/2$, express classical powers through associated photon numbers $P_{p}=N_{p}/T_{p}$ and $P_{SFG}=N_{SFG}/T_{SFG}$, and define $\tau_{s}= 2\pi/\Delta\omega_{s}$ as characteristic duration of photon-pair wavepacket. Then, we note that $\varsigma^{-1}={\tau_{s}N}_{p}/T_{p}$ has the meaning of the number of pump photons within the duration of one photon pair created through SPDC. Since photon pairs are also separated from each other in time, $\varsigma$ is an upper bound of SPDC efficiency. The SPDC efficiency is usually low, of the order of ${10}^{-7}$ per nm of bandwidth for a typical periodically poled lithium niobate waveguide^5^.

The shot noise level is inversely proportional to the square root of the photon number. Then, the shot noise level for SPDC and SFG would be the same if $N_{pair}\approx N_{SFG}$, which leads to a condition for the measurement durations

|  | $\frac{T_{SFG}}{T_{SPDC}}\approx\varsigma\frac{P_{p}^{2}}{P_{s}P_{i}}.$ | (S30) |
| --- | --- | --- |

We see that to achieve the same level of noise, SFG measurements can be many orders of magnitude faster than the accumulation of quantum correlations through SPDC. Importantly, SFG accuracy can be improved and the measurement time reduced by increasing the input laser powers ($P_{s}$ and $P_{i}$), while the pump power for SPDC is limited in order to prevent multi-pair generation.

Next, we estimate how the measurement time scales for increased spectral resolution. Let us consider M frequency intervals within a frequency band $\Delta\omega_{s}$. To keep the same shot noise level, it is necessary to increase the overall integration times for SPDC in each slot by $M$ to get the same photon-pair counts, while there is no change for SFG measurements in CW regime.

We now present estimates based on our device using waveguides 2-3 as an example. We observe that the phase-matching bandwidth is much larger than the spectral filtering applied to the photons. SFG measurement is limited by the speed of the photodiodes and can easily be performed in less than 1 ns. For the input laser powers $P_{s}{\approx P}_{i}\approx800 \mu W$, we detected ~100 nW SFG power corresponding to $\sim{10}^{12}$ photons per second. In quantum regime, our device has a theoretical photon-pair generation rate of 0.93 MHz for $32 \mu W$ pump power, and we find that $\varsigma\sim{10}^{-3}$. Assuming idealised situation of no coupling losses and a detection efficiency of 100%, it would take 6 orders of magnitude longer time for SPDC measurements vs. SFG.

In our experiment, each quantum measurement, such as the one shown in Supplementary Figure S2, required an average integration time of 63 minutes using gated InGaAs avalanche photodiodes with a 100 ns gate window, 11 μs gate period, 8% and 10% detection efficiencies, 10 µs dead time. The largest number of coincidences measured was 2048 for the 2-3 combination, with the corresponding shot noise level of 2.2x10^-2^. If one would use state-of-the-art, free-running superconducting detectors with 93% efficiency and 40 ns dead time^6^ the average measurement time would be reduced down to 0.3 s, considering 0.02 shot noise level for strongest correlations. The quantum characterization of a device, with N modes and high efficiency detectors, requires N(N+1)/2 measurements, corresponding to 4.5 s for 5-mode and 16.5 s for 10 modes. On the other hand, the corresponding SFG measurements can be performed in the nanosecond timescale using standard photodiodes.

Spectral information of the generated photons, for a monochromatic pump at frequency $\omega_{p}$, can be reconstructed using two tunable lasers and scanning their frequencies on the diagonal $\omega_{s}+\omega_{i}=\omega_{p}$. Our SFG measurement required 17 minutes to complete a diagonal scan ($\omega_{s}+\omega_{i}=\omega_{p}$) over a 10 nm bandwidth with a 0.25 nm resolution, being only limited by the slow tuning time of our laser sources that were not optimal for this application. Commercially available tunable lasers can scan 10 nm in 0.1 s with a 1 pm resolution^7^, corresponding to 10000 measurements of 10 μs each. With an SFG power of 100 nw, 5x10^6^ photons are generated in 10 μs, corresponding to a shot noise level of 4.5x10^-4^. The same number of quantum measurement with an upgraded set-up, incorporating high resolution spectrometers or monochromators, and an array of state-of-the-art detectors will take 3000 s, more than 4 orders of magnitude slower, and with quantum shot noise level of 0.02, two orders of magnitude less accurate than with classical measurements.

**Fabrication of the waveguide array**

The waveguides were fabricated on a Z-cut lithium niobate wafer via reverse proton exchange. A titanium mask was used to pattern the channels with a width of 8.5 μm, a coupling region of 2.96 cm in length, and a distance between waveguide centres of 11.6 μm. Proton exchange was performed by immersing the sample in a hot benzoic acid bath creating a 1.85 μm doped layer. Subsequent annealing in air for 7 hours at 328 °C and reverse proton exchange for 8.5 hours at the same temperature were performed.

The poling area is 2 cm long and centered in the middle of the array. The poling pattern was generated by standard electric-field poling, has a poling period $\Lambda=16.07$ μm, and a 50:50 duty cycle. Defects in the poling pattern are located at 1173.11 μm, 4017.50 μm, 7536.83 μm, 13723.78 μm and 19042.95 μm from the beginning of the poling region. S-Bends with a sinusoidal shape and a 5.5 mm length were used at the input and the output of the array to achieve a 127 μm separation between waveguide centres matching the pitch of standard fibre V-groove arrays. The input facet of the chip is polished at an 8° angle to avoid back-reflections into the waveguides.

Splitting ratios of the array were measured at λ=1550 nm for each input waveguide and compared with the prediction of a coupled mode theory for a three waveguide system^8^. The three waveguides are assumed to be identical and with the same coupling coefficient. Theory predictions are calculated for a propagation length $L=3$ cm, where we estimate a bend contribution $\Delta L=400$ μm from numerical simulation of the waveguide fabrication process^9^.

The measured splitting ratios are best fitted, with the same root mean square error RMSE=6.6%, by either of the two coupling rates:

$$C= C_{1}+\frac{2\pi N}{\sqrt{2}L},$$

$$C= C_{2}+\frac{2\pi N}{\sqrt{2}L} ,$$

where $C_{1}=64.4$ m^-1^ and $C_{2}=83.8$ m^-1^, and $N$ is an integer. The estimated set of possible coupling rates is compatible with $C=212.5$ m^-1^ (for $C_{1}$ and $N=1$), which gives a 6.25% deviation from the fabrication target $C=200$ m^-1.^

In the table below we report a comparison between measured splitting ratios and those calculated with a coupled mode theory:

| **WAVEGUIDE INPUT** | **MEASURED SPLITTING RATIOS** | **COUPLED MODE THEORY** |  |
| --- | --- | --- | --- |
| 1 | OUT1= 7.8%  OUT2= 8.2%  OUT3= 84.1% | OUT1= 0.1%  OUT2= 7.9%  OUT3= 91.9% |  |
| 2 | OUT1= 2.3%  OUT2= 87.0%  OUT3= 10.7% | OUT1= 7.9%  OUT2= 84.2%  OUT3= 7.9% |  |
| 3 | OUT1= 87.8%  OUT2= 4.1%  OUT3= 8.2% | OUT1= 91.9%  OUT2= 7.9%  OUT3= 0.1% |  |

TABLE S1. **Comparison between measured splitting ratios of the waveguide array and those calculated with a coupled mode theory.**

Coupling of the pump beam to the adjacent waveguides is found to be negligible due to the smaller mode field diameter. By coupling a laser with λ_p_=775 nm into the first waveguide we measured a 20 dB power suppression on waveguide 2 and a 30 dB power suppression on waveguide 3.

Transmission of the device at λ=1550 nm was monitored during the SPDC characterization by coupling each waveguide with a single-mode fibre and collecting the outputs with a lens with 0.5 NA. The average transmission of the three waveguides is found to be equal to T=31%.

**Design of the poling pattern**

The device under test is based on the recently developed concept of quantum state engineering with specialized poling patterns^10^. The poling geometry was designed to produce an anti-correlated state on waveguides 2-3 while filtering the pump beam - which remains confined to the first waveguide due to the smaller mode field diameter-, corresponding to $\left| \Psi_{23}^{SFG} \right|^{2}= \left| \Psi_{32}^{SFG} \right|^{2}=0.5$, and $\left| \Psi_{n_{s}n_{i}}^{SFG} \right|^{2}=0$ otherwise. Defects locations are calculated with an optimization algorithm^10^ for a coupling rate $C=200\pm20$ m^-1^ and a 16.07 µm poling period.

We observe that the fabricated device successfully generates a nearly anti-correlated state on the desired waveguides with a suppression of two-photon contributions on the single spatial channels. The presence of the non-zero terms$\left| \Psi_{12}^{SFG} \right|^{2}, \left| \Psi_{21}^{SFG} \right|^{2}$ reduces the fidelity between the desired state and the one generated by the device to F=0.67±0.01. This discrepancy from the target state could be caused by non-uniformities in the refractive index profile of the waveguides along the propagation direction, imperfections in the poling pattern, and deviation from the target coupling rate.

**SFG-phase measurements**

We describe, as an example, the procedure used for phase measurements for the case of waveguides 2-3. An equivalent procedure was used for all the other waveguide combinations. Let us call, with reference to Figure 3a in the main text, $\Delta\phi_{s}\left( \Delta\phi_{i} \right)$the phase difference at the input of the array between signal(idler) beam injected into waveguide 2(3) and signal(idler) beam injected into waveguide 1(1) when no voltage is applied to the phase modulator. Thermal and mechanical fluctuations are negligible in the given acquisition time and the two phase differences are assumed to be constant.

The goal of the characterization is to measure the value $\theta^{SFG}-\theta^{s}-\theta^{i}$. $\theta^{SFG}$ is the relative phase between

the sum-frequency fields generated from the combinations of signal-idler beams injected into waveguides 2-3 and signal-idler beams injected into waveguides 1-1 introduced by the array at the output of waveguide 1. $\theta^{s}(\theta^{i})$ is the phase difference between the signal(idler) beam injected into waveguide 2(3) and the signal(idler) beam injected into waveguide 1(1) introduced by the array at the output of the same waveguide. When a phase modulation with frequency $f$ is applied to the first channel at the input, the three generated interference patterns can be expressed as

$$\left| E_{23}^{SFG}+E_{11}^{SFG} \right|^{2}\propto\cos\left( \theta^{SFG}+\Delta\phi_{s}+\Delta\phi_{i}-4\pi ft \right)+constant terms,$$

$$\left| E_{2}^{s}+E_{1}^{s} \right|^{2}\propto\cos\left( \theta^{s}+\Delta\phi_{s}-2\pi ft \right)+constant terms,$$

$$\left| E_{3}^{i}+E_{1}^{i} \right|^{2}\propto\cos\left( \theta^{i}+\Delta\phi_{i}-2\pi ft \right)+constant terms.$$

To obtain the desired values, the acquired oscilloscope traces for signal and idler beams are fitted with the two functions $y_{s}=a_{s}\cos\left( c_{s}-2\pi ft \right)+d_{s}$ and $y_{i}=a_{i}\cos\left( c_{i}-2\pi ft \right)+d_{i}$. A fast fourier transform analysis of the oscilloscope trace for SFG reveals that the signal is made of a fast component oscillating with frequency $2f$and a slow component with frequency $f$ due to the interference of sum-frequency fields generated from combinations other than waveguides 2-3 and waveguides 1-1 (namely, waveguides 1-2 and waveguides 1-3). Hence, the SFG trace is fitted with the function $y_{SFG}=a_{1}\cos\left( c_{1}-2\pi ft \right)+a_{2}\cos\left( c_{2}-4\pi ft \right)+d .$The desired phases are finally calculated as $\theta^{SFG}-\theta^{s}-\theta^{i}=c_{2}-c_{s}-c_{i}.$ Uncertainties in the measurements are obtained from the confidence bounds in the least squares fitting procedure.

**Calculation of the Schmidt number from SFG-phase measurements.**

The degree of entanglement in the predicted state is calculated in a non-degenerate case for two fixed frequencies $\omega_{s}, \omega_{i}$ by expressing the biphoton state as

$$\left| \Psi\right\rangle_{pair}= \sum_{n_{s}n_{i}} \Psi_{n_{s}n_{i}}\hat{a}_{n_{s}}^{\dagger}\left( \omega_{s} \right)\hat{a}_{n_{i}}^{\dagger}\left( \omega_{i} \right)\left| 0 \right\rangle.$$

The amplitudes of the wavefunction elements $\Psi_{n_{s}n_{i}}$are calculated from the results of SFG-power measure-

ments, while the relative phases are calculated from the results of SFG-phase measurements for the two fixed wavelengths used in the characterization. The Schmidt number S is obtained through the Schmidt decomposition for a bipartite system

$$\Psi_{n_{s}n_{i}}= \sum_{j} \sqrt{S_{j}}U_{jn_{s}}V_{jn_{i}},$$

as

$$S=\frac{1}{\sum_{j} S_{j}^{2}} .$$

**Photon pair generation rates and wavefunction square moduli from SPDC measurements**.

Absolute photon pair generation rates were calculated for each input combination $n_{s}, n_{i}$ as

$$\frac{dN_{pair}}{dt}=\frac{C_{n_{s}n_{i}}}{\Delta T \mu_{n_{s}}\mu_{n_{i}}\eta_{1}\eta_{2}} ,$$

where $\Delta T$is the acquisition time, $\mu_{n_{s}}, \mu_{n_{i}}$ are the total transmissions of the single channels (including paths from waveguides to detectors), and $\eta_{1}, \eta_{2}$ are the quantum efficiencies of the two detectors. $C_{n_{s}n_{i}}$is the total number of counts in a time window equal to 2 FWHM centered around the coincidence peak acquired with the time tagging module. Accidental counts were measured in an equal time window away from the peak and subtracted from this value.

To take into account fluctuations in the pump power (≈ 15 %) during the acquisition time, coincidence values were rescaled by using the average total number of counts measured from waveguide 2 as a common reference for the average power. Squared relative amplitudes of the wavefunction elements were calculated as

$$\left| \Psi_{n_{s}n_{i}}^{SPDC} \right|^{2}= \frac{{C_{n_{s}n_{i}}}/{\mu_{n_{s}}\mu_{n_{i}}}}{\sum_{n_{s}n_{i}} [{C_{n_{s}n_{i}}}/{\mu_{n_{s}}\mu_{n_{i}}}]} .$$

## **SFG characterisation data**

Figure S1 shows the SFG efficiencies as a function of the wavelengths of signal and idler lasers for all input combinations. SFG power is measured from waveguide 1. Corresponding measurements of photon pair generation via SPDC are shown in Figure S2 for a pump wavelength $\lambda_{p}=775$ nm, and power $P_{p}=32\pm5$ μW. Time bin width is 82 ps. Figure S3 shows the complete set of interferometric measurements used for phase reconstruction. The phases of the different elements $\Psi_{ij}$ were measured relative to $\Psi_{11}$.


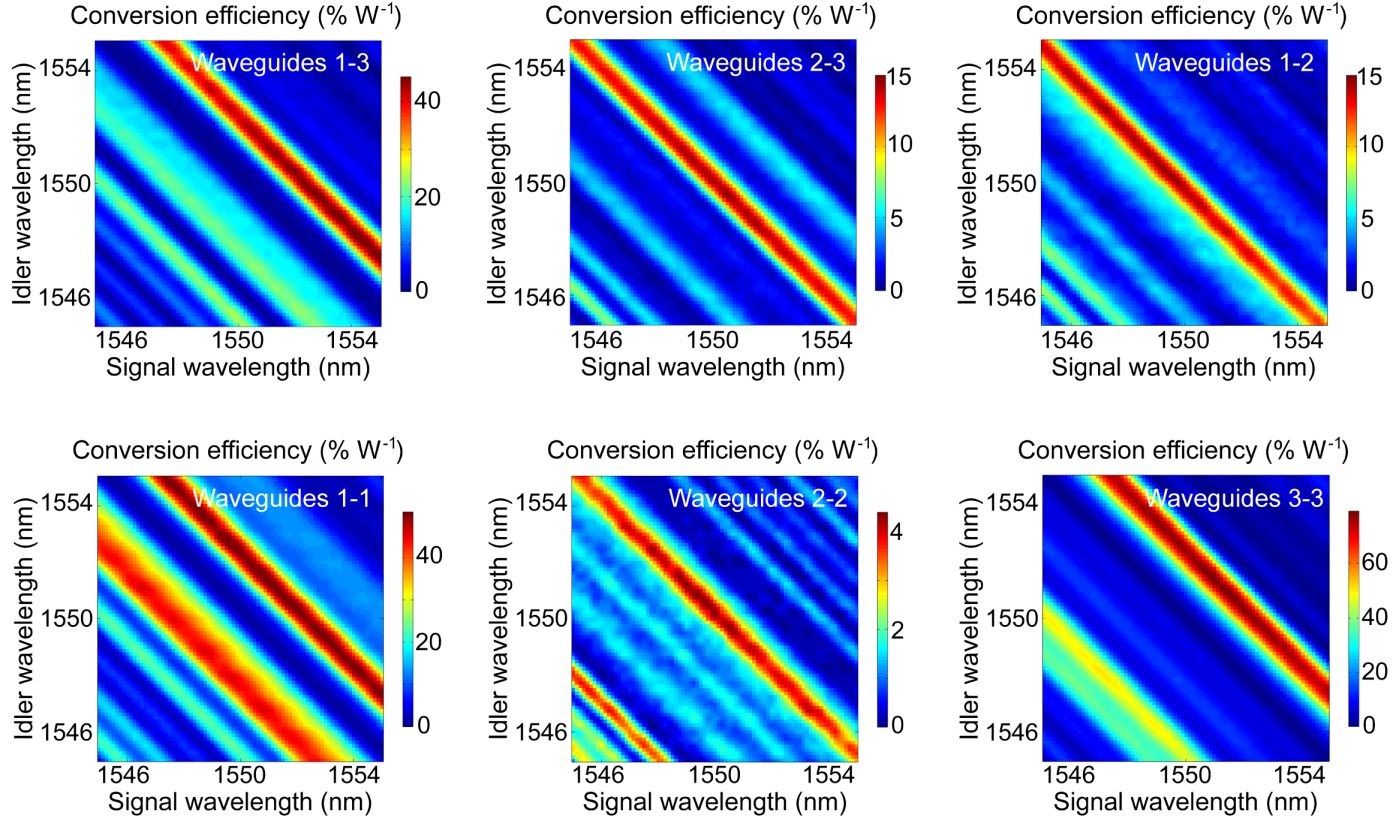


FIGURE S1. **SFG Measurements.** Measured classical sum-frequency conversion efficiency from waveguide 1 as a function of signal and idler wavelengths for all input combinations.


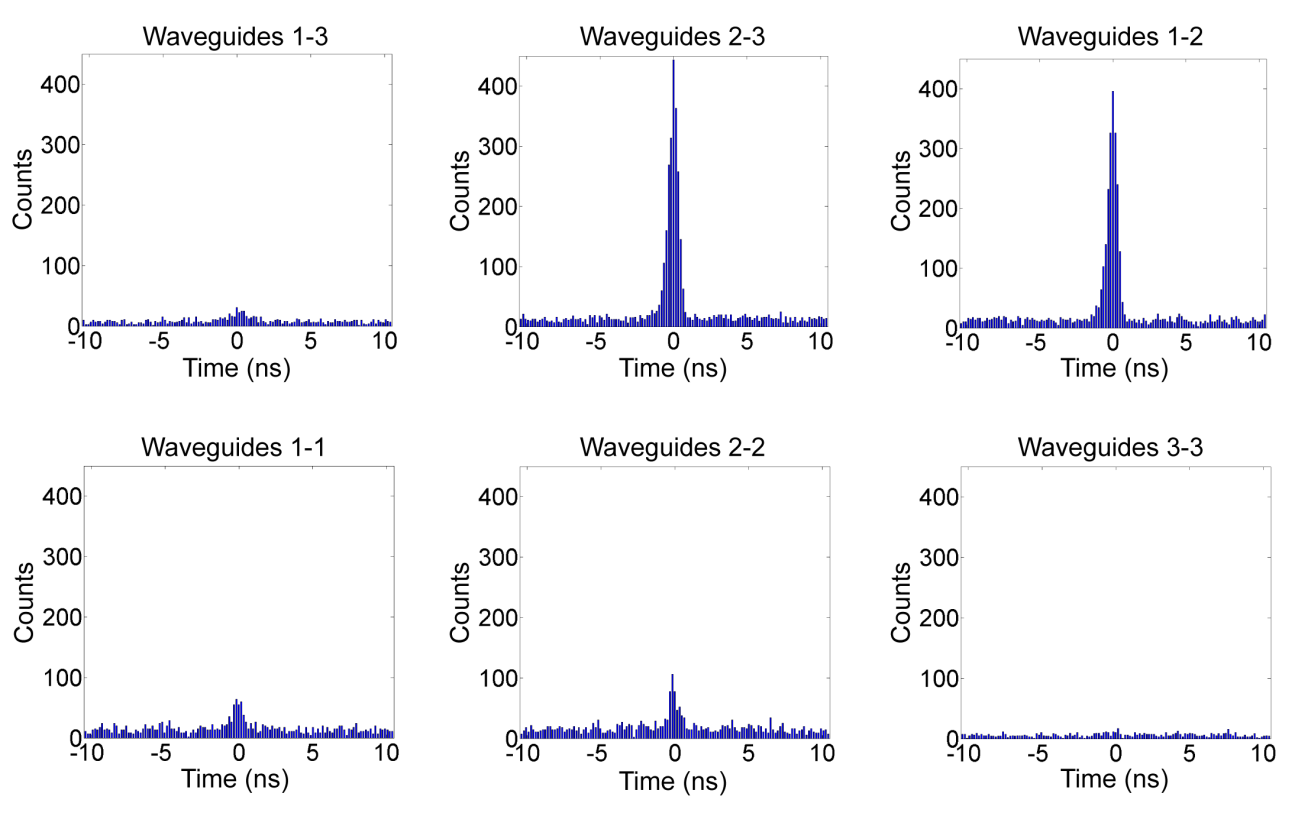


FIGURE S2. **SPDC measurements.** Coincidence measurements for all output combinations when a pump beam with wavelength $\lambda_{p}$ = 775 nm, and power P_p_ = 32± 5 µW is coupled to waveguide 1.


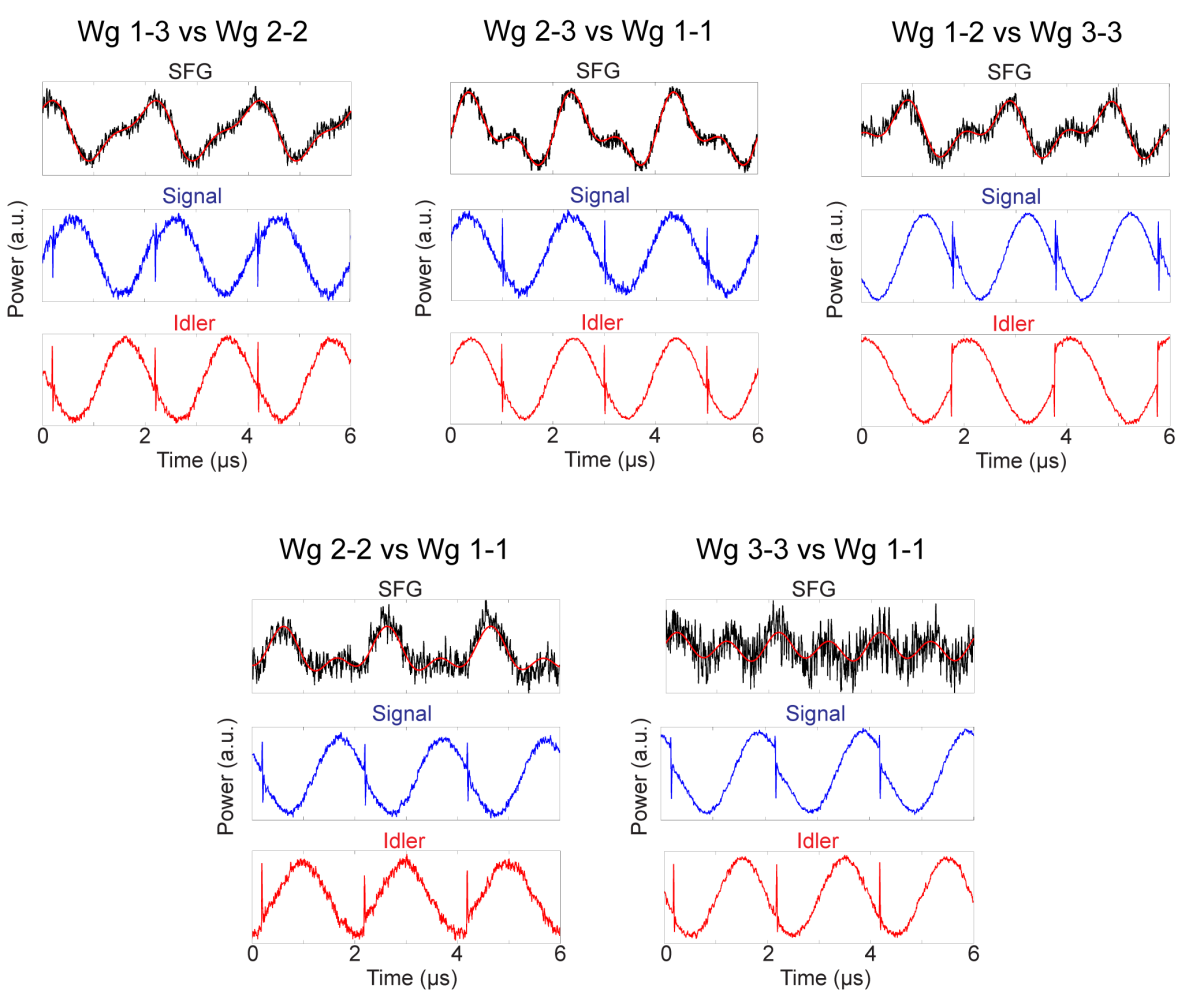


FIGURE S3. **Phase measurements.** Oscilloscope traces obtained by collecting the beams with three different photodiodes for all input combinations. For each combination there are three traces that are used to measure the relative phase between the wave function elements $\psi_{ij}$ and $\psi_{11}$. Solid red line is the theoretical fit.

## **Squared relative amplitudes of the wavefunction elements from SFG and SPDC measurements**

| **WF Element** | **SPDC** | **SFG** |
| --- | --- | --- |
| $\left\vert\psi_{13} \right\vert^{2},\left\vert\psi_{31} \right\vert^{2}$ | $0.013\pm0.003$ | $0.033\pm0.005$ |
| $\left\vert\psi_{23} \right\vert^{2},\left\vert\psi_{32} \right\vert^{2}$ | $0.229\pm0.013$ | $0.218\pm0.005$ |
| $\left\vert\psi_{12} \right\vert^{2},\left\vert\psi_{21} \right\vert^{2}$ | $0.212\pm0.015$ | $0.195\pm0.007$ |
| $\left\vert\psi_{11} \right\vert^{2}$ | $0.040\pm0.010$ | $0.040\pm0.008$ |
| $\left\vert\psi_{22} \right\vert^{2}$ | $0.046\pm0.007$ | $0.059\pm0.005$ |
| $\left\vert\psi_{33} \right\vert^{2}$ | $0.006\pm0.002$ | $0.009\pm0.004$ |

TABLE S2. **Squared relative amplitudes of the wavefunction elements.** Errors for SPDC take into account the poissonian statistics of the detection process and uncertainties in transmission measurements. Errors for SFG take into account uncertainty in optical power measurements. The fidelity between the two matrices is $\left( 99.28\pm0.31 \right)\%$.

**References**

1 Born M, Wolf E. *Principles of Optics: Electromagnetic Theory of Propagation, Interference and Diffraction of Light.* Cambridge University Press: Cambridge, 1999.

2 Poddubny AN, Iorsh IV, Sukhorukov AA. Generation of Photon-Plasmon Quantum States in Nonlinear Hyperbolic Metamaterials. *Phys Rev Lett* 2016; **117**: 123901-6.

3 Sukhorukov AA, Solntsev AS, Kruk SS, Neshev DN, Kivshar YS. Nonlinear coupled-mode theory for periodic plasmonic waveguides and metamaterials with loss and gain. *Opt Lett* 2014; **39**: 462-5.

4 Chen PY, McPhedran RC, De Sterke CM, Poulton CG, Asatryan AA *et al.* Group velocity in lossy periodic structured media. *Phys Rev A* 2010, **82**: 053825-36.

5 Tanzilli S, De Riedmatten H, Tittel W, Zbinden H, Baldi P, *et al.* Highly efficient photon-pair source using periodically poled lithium niobate waveguide. Electron. Lett. 2001; **37**: 26-28.

6 Marsili F, Verma VB, Stern JA, Harrington S, Lita AE, *et al.* Detecting single infrared photons with 93% system efficiency. *Nat Photonics* 2013; **7**: 210-214.

7 Yenista Optics company. Tunics T100R tunable laser Data Sheet [PDF on internet]. Lannion:France, [cited 12 October 2017]. Available from: https://yenista.com/Tunics-T100R,29.html.

8 Paspalakis E. Adiabatic three-waveguide directional coupler. *Opt Commun* 2006; **258**: 30-34.

9 Lenzini F, Kasture S, Haylock B, Lobino M. Anisotropic model for the fabrication of annealed and reverse proton exchanged waveguides in congruent lithium niobate. *Opt Express* 2015; **23**: 1748-1756.

10 Titchener JG, Solntsev AS, Sukhorukov AA. Generation of photons with all-optically-reconfigurable entanglement in integrated nonlinear waveguides. *Phys* *Rev* *A* 2015; **92**: 033819.
